# Coexistence of thought types as an attentional state during a sustained attention task

**DOI:** 10.1038/s41598-023-28690-1

**Published:** 2023-01-28

**Authors:** Kazushi Shinagawa, Yu Itagaki, Satoshi Umeda

**Affiliations:** 1 Department of Psychology, Keio University, 2-15-45 Mita, Minato-Ku, Tokyo, 108-8345 Japan; 2 Japan Society for the Promotion of Science, Kojimachi Business Center Building, 5-3-1, Kojimachi, Chiyoda-ku, Tokyo, 102-0083 Japan; 3 Keio University Global Research Institute, 2-15-45 Mita, Minato-ku, Tokyo, 108-8345 Japan

**Keywords:** Psychology, Human behaviour

## Abstract

Some studies have asked participants about attentional state on a scale from on-task to off-task, which set the middle option as attention focused on both, including the possibility of the coexistence of thoughts. In addition, studies using multidimensional probes explicitly assumed coexistence within spontaneous thoughts and task-focus dimensions. Although several studies have assumed the coexistence of some thought dimensions, none has explored whether these are different types of thoughts (task-focus, mind-wandering, task-related, external stimuli-related). To examine whether this coexistence of thought types occurred, we used thought probes to determine the degree of immersion in each. The participants responded to probes presented at random during a sustained attention task. The results revealed a mixture of thought types in many self-reports. In addition, the state of attentional allocation behind self-reports was estimated using the hidden Markov model. We observed the following attentional states: task-focused, task-unrelated, task-related, external stimuli-focused, and task-focused—but also focused on other thoughts. These results suggest that individuals can simultaneously allocate attention to thought types and discriminate between reporting. In some cases, probe options should also be considered for this coexistence. We also examined the relationship between self-reports and behavioral indexes, and discussed the necessity of separately measuring the degree of immersion for each thought type.

## Introduction

People often focus on thoughts unrelated to the here-and-now^1^. This experience is referred to as mind-wandering (MW), and it occupies a large portion of time in our daily lives^2^. Many studies have used probe-caught methods, which ask participants about the content of their experience at random times during a sustained attention task^3,4^. As the responses to probes are directly interpreted as an individual’s MW tendencies, the options of probes need to be decided carefully and are still being discussed^5^.

In many studies, probe-caught methods force participants to choose whether they have focused on the task or MW^4^. Recent research has suggested that such binary options can bias participants' responses, and that ratings between them are needed. Seli et al.^6^ conducted an experiment in which participants were asked whether they focused on the task or MW with a dichotomous and five-point Likert scale in everyday life. With the dichotomous method, 40% of the probes were reported as MW, whereas, with the five-point Likert scale, only 12–20% showed a high degree of MW. This result suggests that the self-reports of MW to dichotomy probes may have included uncertainty and that including the multiple options between task and MW is preferred for more accurate measurement of MW.

While the gradient options between the task and MW have been employed, many studies have included other thought dimensions. Task- and external-stimulus relatedness were the two criteria used in the study to divide being off-task thought into four categories^1^. In this measure, when the thought contents are task-related and perceptually guided, the participants focus on the task. One type of thought classified as off-task is when the content of the thought is self-generated and task-related. The state in which participants consider the purpose of the task is one example, which is different from the state in which they are focused on the task. The thought contents depend on external stimuli and are unrelated to task are also classified as one type of off-task thought, as when a participant is distracted by a phone call. Finally, there is a state of complete detachment from external stimuli and tasks, and the contemplation of future plans. As in this definition, many studies have included task-related and external stimuli-related thoughts in their probes, in addition to task-focused and task-unrelated thoughts (TUT)^7–10^. According to Robison et al.^5^, participants may equate task-related thoughts with task-focused thoughts and TUTs. The authors also suggested that the options to catch MW should include at least task-focused thoughts, task-related thoughts, and TUTs. More recently, it has been shown that asking about the type of thought (such as task- or external stimuli-related, daydream, or current concern) is more valid and reliable than including the gradation between task and MW^11^.

Previous studies have asked participants to report the one thought type they were engaged in from the options^7,12,13^. However, individuals may simultaneously allocate attention to multiple thought types. Research on cognitive resources has suggested that different mental resources represent visual and auditory perceptions^14,15^. Thus, when the task and MW require different modality resources, they can be simultaneously maintained. For example, participants respond to auditory stimuli while storing representations related to TUTs in unused visual resources. Research on divided attention also suggests that when the demands of one task are insufficient to consume all mental resources, leftover resources are allocated to a secondary task^15,16^. From this perspective, the coexistence of attentional allocation to different thought types may exist within the same modality. One study examining the relationship between modalities used in the main task and MW showed that visual MW tended to be more frequent when visual stimuli were included in the main task than when auditory stimuli were included^17^. They used a two-back task as the primary task, which requires high concentration and maintenance of representations. The increase in MW in the same modality as the main task suggests that participants may concentrate on the task and maintain their MW regardless of the modality.

MW research has also supported the coexistence of MW and tasks. The resource hypothesis suggests that the main task and the MW consume the same cognitive resources^3^. Therefore, there may be a state in which participants are immersed in MW while conducting the main task as long as resources are available. An electroencephalography study showed that the component reflecting the allocation of attention to a task was reduced when participants focused on the MW^18^. The authors suggested that this result might have been due to the simultaneous allocation of attentional resources to the task and the TUTs. A component reflecting the allocation of attention to the task was observed during MW in other studies, supporting this hypothesis^19,20^. Additionally, studies using probes that ask for participants’ thoughts gradually set the middle option as attention focused on both the task and MW^11,21^, including the possibility that the participants reported the coexistence of these thoughts. However, participants in these studies reported the main thoughts in their attentional allocation and did not mention other resources. Thus, this method could not examine whether other objects of attention simultaneously existed when they were focused on the task or MW. The option, such as “mostly on task unrelated concerns,” may be reported when the main thought was MW. Participants may also provide the same answer when they marginally focus their main thoughts on MW and the task.

Recent research has explicitly assumed the coexistence of certain thoughts. S studies using multidimensional experience sampling (MDES) asked participants to report their focus on some dimensions, including tasks and TUTs^22–24^. These studies used various dimensions of spontaneous thought and task focus to capture complex thought states. This study focused on off-task occurrence patterns and task-relevance dimensions (task-focused, task-related, external stimuli-related, and task-unrelated). Explicitly examining the coexistence of various processing types of thoughts may help more accurately investigate the tendency of participants’ attentional states. Therefore, we investigated whether thought types coexist by using thought probes to determine the degree of immersion in each thought type.

This study examines whether coexistence is a meaningful state of attention. Some studies estimated the attentional states behind behavioral indices or self-reports using the hidden Markov model (HMM). The HMM is a Markov process with unobserved states. A Markov process is a stochastic process, in which the probability of the following state depends only on the current state. Previous research has assumed that unobserved states are attentional allocation (task-focused/TUT-focused). These studies also assumed that attentional states probabilistically produce self-reports and reaction times. Bastian and Sackur^25^ used the HMM on time-series data of reaction times to estimate participants’ attentional states between task-focused and MW states. Zanesco et al.^26^ used the HMM to examine distinct and separable patterns of self-reports as an attentional state. They used probes that included six-level continuum options from on-task to off-task, and estimated three states of attentional allocation behind the self-reports. The estimated states were task-focused, and TUT-focused, and between the two, rather than a dichotomy. In this study, we used probes assuming the coexistence of multiple thoughts and examined the type of attentional state estimated from self-reports using HMM.

Although many indexes are related to MW, behavioral variability has been widely recognized, such as response time variability (RTV: standard deviation [SD]/mean). The previous study also suggested that behavioral variability is an on-time indicator of MW in some tasks^27^. However, subsequent research has shown that the brain regions influencing self-reported TUTs and RTV differ, suggesting more complex correspondence^28^. While the attentional state has been estimated behind self-reports and reaction times^25,26^, the relationships between estimated states from these have not been investigated. Therefore, the present study also aimed to provide insight into the relationship between self-reported MW and reaction times variability by comparing the attentional state from self-reports and RTV.

## Methods

### Participants

This study recruited 31 university students (12 males and 19 females; mean age = 21.4, SD = 1.43; range = 19–25 years) at Keio University. None of the participants had visual or hearing impairments or a history of mental or neurological disorders. No statistical analyses were conducted to predetermine the sample sizes, but our sample sizes were similar to those generally employed in the field. As we conducted a correlation analysis and an analysis of variance (ANOVA) to detect the relationships between self-reports and behavioral measures, the effect size assumed in these analyses was moderate^26,29^. Under these conditions (*r* = 0.5, *α* = 0.8; *f* = 0.3, *α* = 0.8), the required sample size was *n* = 28.25 and 31.27. Additionally, the results of this study showed an effect size close to the assumed effect size. Therefore, the sample size used in this study is considered reasonable. This study was approved by the Keio University Research Ethics Committee (No. 18006) and was conducted per the Declaration of Helsinki. All participants provided prior written informed consent.

### Procedures

Participants were required to wear earplugs and maintain the distance of their faces and bodies from the screen while performing the tasks. The background for the task was black, and the instruction text was white. We presented each stimulus, recording responses and correcting the answers to questionnaires using a personal computer (OptiPlex 7040, Dell) with PsychoPy for stimuli presentation and response recording^30^.

The sustained attention to response task (SART) was used as the main task to measure the tendency to engage in spontaneous thoughts. Participants were required to press the space key as quickly as possible when the non-target stimuli (numbers 1–4, 6–9) were presented and waited for the target stimuli (the number 5) to disappear without doing anything. Numbers 1 through 9 were randomly presented for 350 ms, and the interval between stimuli was 1150 ms. We conducted 20 blocks, each consisting of 45 trials as the main task and 15 trials as practice.

We sampled the spontaneous thoughts during the task by presenting one thought probe randomly within each block (the minimum interval between probes was 15 trials). In the probes, the participants were required to rate immersion level in task-focused thoughts (the degree of concentration on the task) and task-related thoughts (thoughts about task-related information, such as task performance and time spent on the task). They also rated external stimuli-related thoughts (thoughts about external stimuli, such as light and sound, and physical sensations, such as hunger and thirst) and TUTs (task-unrelated and self-generated thoughts, such as planning future events). All these ratings were on a five-point scale ranging from 0%–100% by 25% (0%, 25%, 50%, 75%, 100%). The left end of the scale was 0%, and the right end was 100%. We explained that immersion is how participants allocate their attention to each type of thought. Questions about the immersion level for each thought type were asked on a single screen, from top to bottom: task-focused, task-related, external stimuli-related, and task-unrelated thought. The mouse cursor appeared at the center of the screen when the thought probe was presented to prevent response bias. Furthermore, we set no initial position on the scale of each thought type, and a pointer appeared when the participant made the first response. The participants answered the thought probes by moving a pointer along a horizontal scale with a left mouse click. We instructed the participants to keep the total immersion level below 100% and report a state of mind blanking (in which the participant was not concentrating on any of the above thoughts) by responding 0% regardless of thought type. We programmed a task in which participants could have their responses add up to more than 100%. However, only one participant provided more than 100% of the responses. These responses were interpolated using the missing values.

### Statistical analysis

#### Effects of the thought types on response time variability

We explored the effect of immersion in each thought on task performance using RTV as the TUT index, referring to studies exploring the impact of MW on behavioral indices^25,31^. Although previous studies have not examined the interaction between thought types, the effect on behavioral indices may differ depending on how thoughts consume attentional resources. For example, the effect on the behavioral index may differ when attention is allocated to tasks with and without TUTs. Therefore, we conducted a multiple regression analysis with RTV as the dependent variable and the interaction between immersion in the task and TUTs as independent variables. We calculated the mean reaction times and RTV for each probe response using five trials preceding the probes to examine the relationships between thought types and behavior measures. The participants responded correctly to the go trial before probe presentation. Furthermore, correlations between variables are likely to be higher in multiple regression analyses that involve interactions. We conducted centering to avoid multicollinearity by subtracting the mean value from each independent variable. This process is commonly recommended to mitigate multicollinearity between the independent variables and constructed interactions. Task-related and external stimuli-related thoughts were excluded from the linear analysis because there were not enough trials of immersion of 100% for the analysis. All analyses were conducted using the open statistical software R (R 4.1.0)^32^.

#### Estimation of attentional states using hidden Markov models

We used an HMM to estimate the attentional states behind self-reports and behavioral measures. As mentioned above, HMM assumes that a Markov chain with a finite number of latent (unobservable) states generates observed data (i.e., self-reports for probes/RTV). Previous MW studies have recognized latent states as attentional states. This study also estimated the time-varying attentional state that generates RTV and self-reporting.

To estimate the states behind the self-reports and behavioral measures, we used the time-series data of the immersion for each thought type (20 probes in total) and RTV as input (observed sequence) for the HMM. To calculate the time series of RTV, we used five correct go trials, starting from the first trial and advancing by one trial each time until the end. The time-series data for each participant were then concatenated to estimate attentional states. We used multivariate (four variables, immersion level of each thought) and univariate time-series data for the HMM with self-reports and RTV, respectively. The self-reported data were assumed to follow a multinomial distribution because the immersion level ranged from 1–5 (0%–100%) for each thought type. For RTV, a normal distribution was assumed because it is an index related to the reaction time. We used the “depmixS4”^33^ package in the R 4.1.0 to implement the HMM. In this package, the model parameters were estimated using the expectation–maximization algorithm. This algorithm iteratively maximizes the joint log-likelihood of the parameters, given the observations and states. The number of hidden states must be determined prior to the estimation. A previous study determined this number using Bayesian information criterion (BIC)^26^. BIC, calculated for each HMM with a different number of states, is recognized as a criterion for model selection. We compared up to eight models, including one to eight states, considering the states immersed in only one thought, and the coexistence of some thoughts. We also compared eight models when HMMs were applied to the RTV. After state estimation, we explored the relationship between attentional state and other measures. First, a one-way analysis of variance was conducted on RTV, factoring across the estimated states to examine the trends in the estimated states of attention. Second, correlation analyses with estimated states from self-reports and behaviors were conducted to examine how estimated states relate to other indices. Because we conducted multiple comparisons, the correction analyses were corrected using the false discovery rate method to control for the probability of false positive detection.

## Results

### Results of the SART

In the self-reports during the SART, one participant reported experiencing TUTs despite 0% immersion in TUTs. Due to misunderstanding the instructions, this participant was excluded from the analysis. Moreover, one participant who showed a bimodal distribution of reaction times, with some extremely fast and slow reaction times, was also excluded because the data might not have been obtained correctly. Finally, one participant whose probes were not presented because of a system error during the task was excluded. Ultimately, we included 28 participants in the analysis.

First, we show the rating trends for the probes in this study. Table 1 shows the average response of the immersion in each thought for all participants. No participants reported being 100% immersed in external stimuli-related thoughts. Similarly, 100% immersion in task-related thoughts was obtained once. Of all the self-reports, 11.5% indicated 100% immersion, and only 4.7% were occupied by task-focused thought. Mind-blanking was observed 21/560 times (3.62%). Because many participants did not show mind-blanking, we did not calculate the reaction time.

**Table 1:** The average number of reports for each level of immersion. The mean percentages are in parentheses and the total number of probe is 20 per participant.

		0%	25%	50%	75%	100%
Task-focus	Mean	6.00 (.30)	6.46 (.32)	5.86 (.29)	5.04 (.25)	2.70 (.14)
	SD	4.93 (.25)	4.76 (.23)	2.85 (.14)	3.44 (.17)	1.89 (.09)
Task-related	Mean	9.88 (.49)	8.74 (.44)	2.85 (.14)	2.14 (.11)	1.00 (.05)
	SD	5.68 (.28)	4.41 (.22)	2.21 (.11)	1.07 (.05)	0.00 (.00)
External stimuli	Mean	12.39 (.62)	6.17 (.31)	2.50 (.13)	1.50 (.08)	NA
	SD	4.95 (.25)	3.19 (.16)	1.85 (.09)	0.53 (.02)	NA
TUT	Mean	12.29 (.61)	4.73 (.24)	2.65 (.13)	2.33 (.12)	8.00 (.40)
	SD	4.63 (.23)	2.44 (.12)	1.79 (.09)	1.87 (.09)	5.66 (.28)

SD = standard deviation; TUT = task-unrelated thought.

Next, we investigated the probability that participants reported only one type of thought dimension. That is, the number of responses in which immersion in a particular type of thought was 25% or more and the other types of thought were 0%. The number of reports that included only one type of thought was 79/560 (14.1%). The number of responses with two or more levels of immersion, that is, the coexistence of multiple thoughts was out of 458/560 (81.8%). Among these, 50% of the attention allocated to the two thoughts was reported of 61/560 times (10.9%). The number of single thoughts was lower than expected, and most were a mixture of multiple thoughts. See Supplementary Table S1 for a detailed pattern of self-reports.

### Regression analysis of the SART

The mean reaction time and RTV for each level of immersion in thought types are shown in Tables 2 and 3. No exclusion criteria were set for calculating RT or RTV for each individual or group. However, since responses with no sample or a sample size of 1 could not theoretically be calculated, the RT and RTCV for 100% of task-related and external stimuli-related thoughts were not calculated at the group level.

**Table 2:** Average reaction times per response to the probes.

		0%	25%	50%	75%	100%
Task-focus	Mean	0.330	0.329	0.320	0.313	0.326
	SD	0.055	0.060	0.043	0.042	0.032
Task-related	Mean	0.326	0.316	0.332	0.330	NA
	SD	0.050	0.044	0.045	0.045	NA
External stimuli	Mean	0.316	0.325	0.319	0.357	NA
	SD	0.035	0.045	0.064	0.091	NA
TUT	Mean	0.318	0.325	0.321	0.300	0.300
	SD	0.039	0.051	0.058	0.050	0.064

SD = standard deviation; TUT = task-unrelated thought.

**Table 3:** The response time coefficient of variability per response to the probes.

		0%	25%	50%	75%	100%
Task-focus	Mean	0.316	0.190	0.144	0.135	0.152
	SD	0.248	0.100	0.053	0.078	0.126
Task-related	Mean	0.199	0.156	0.203	0.202	NA
	SD	0.100	0.103	0.240	0.171	NA
External stimuli	Mean	0.178	0.199	0.191	0.234	NA
	SD	0.091	0.153	0.230	0.108	NA
TUT	Mean	0.164	0.178	0.199	0.253	0.437
	SD	0.085	0.117	0.144	0.110	0.203

SD = standard deviation; TUT = task-unrelated thought.

**Figure 1:**
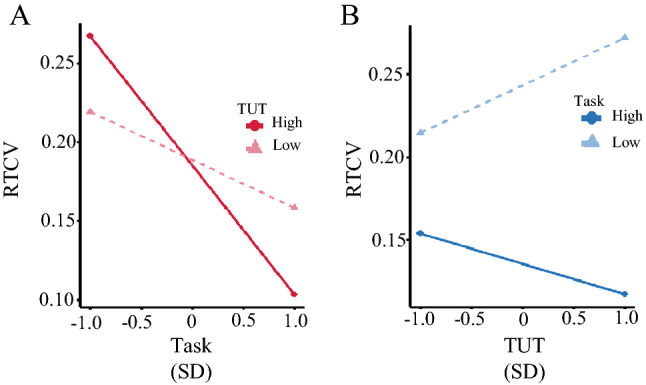
The simple slope analysis of the interaction between task-focus and task-unrelated thought (TUT) on the response time coefficient of variability (RTV). (**A**) The dark and light red lines indicate when responses to TUTs are relatively high and low, respectively. The horizontal axis shows the level of task concentration, and the vertical axis shows the RTV. When the immersion in TUTs was high, the effect of RTV on the task was substantial, whereas when it was relatively low, the effect of task concentration on the RTV was small. (**B**) The dark and light blue lines indicate when responses to task focus are relatively high and low, respectively. The horizontal axis shows the level of TUT immersion, and the vertical axis shows the RTV. While RTV was increased with TUT level when the participant did not focus on the task, when the concentration was high, there was no significant effect of TUT on RTV.

In this study, we conducted a multiple regression analysis to investigate the interactive effects of thought type on behavior. The results are presented in Table 4 (*adjusted R^2* = 0.093, *F*[ 3, 281] = 10.72, *p* < 0.001). In this study, we used the variance inflation factor (VIF) as a measure of multicollinearity in multiple regression analyses. This was calculated by regressing an independent variable against every independent variable in the model. Consequently, the maximum value of the VIF was 1.669, below the strict criterion of 4, and is therefore unlikely to contain multicollinearity, which affected the results. Because the interaction between immersion in the task and TUTs was significant, we conducted a simple slope analysis (Fig. 1). Continuous data are shown separately for illustration. The relatively high state is designated as mean + 1 SD and the low state as mean −1 SD. First, we examined the change in the effect of task concentration on RTV depending on the level of immersion in the TUTs (Fig. 1A). The results indicated that when immersion in TUTs was high, RTV decreased significantly with higher levels of task concentration (*β* = −0.078, *SE* = 0.018, *p* < 0.001). In contrast, when immersion in TUTs was low, task concentration had little effect on RTV (*β* = −0.03, *SE* = 0.01, *p* = 0.03). We also examined the effect of TUT immersion on task concentration (Fig. 1B). The results indicated that RTV increased with the TUT level when the participant did not focus on the task (*β* = 0.026, *SE* = 0.013, *p* = 0.04). Subsequently, when the concentration on the task was high, there was no significant effect of TUT on RTV (*β* = −0.03, *SE* = 0.03, *p* = 0.23).

**Table 4:** Results of the multiple regression analysis.

Variable	Partial regression coefficient	SE	Beta	VIF
Task-focus	−.053	.011	−.30**	1.265
TUT	−.002	.015	−.009	1.669
Task-focus: TUT	−.027	.013	−.142 *	1.480

SE = standard error; VIF = variance inflation factor; .01: "**", .05: "*".

We analyzed RTV as the dependent variable and the interaction between immersion in the task and TUTs as independent variables.

### Results of the hidden Markov model (self-reports)

**Figure 2:**
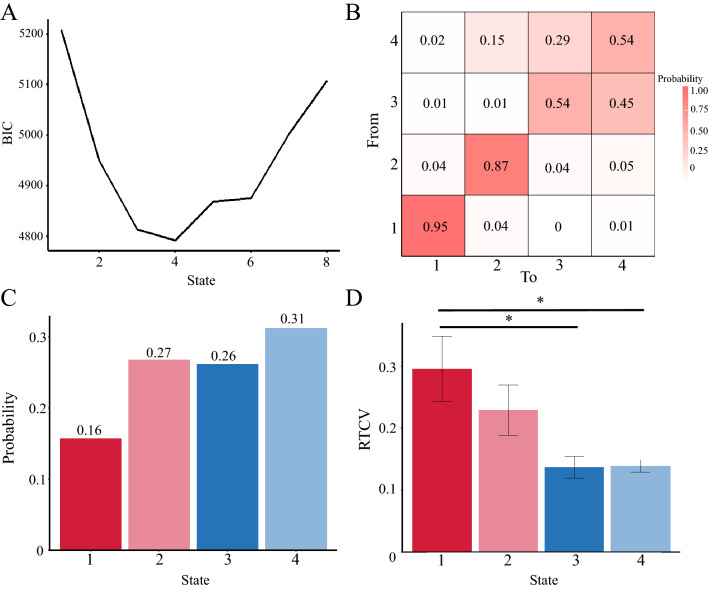
The results of the hidden Markov model. (**A**) The Bayesian information criterion (BIC) for each number of states. The BIC decreases up to four states but increases after that point. (**B**) Transition probability between states. A color and numerical value indicate the transition probabilities from the state on the vertical axis to those on the horizontal axis. The darker color indicates a higher transition probability. (**C**) Appearance probability during the task by states. The horizontal axis indicates the state, and the vertical axis shows the probability. The probability of State 1 is the lowest, and the probability of State 2 is the highest. (**D**) The response time variability per each state. The horizontal axis indicates the state, and the vertical axis shows the RTV. The RTV during State 1 is significantly greater than in States 3 and 4. Each state was defined as TUT, unfocused, task-focused, and mixed. The error bar and asterisks indicated standard error and p ≤ .01.

**Figure 3:**
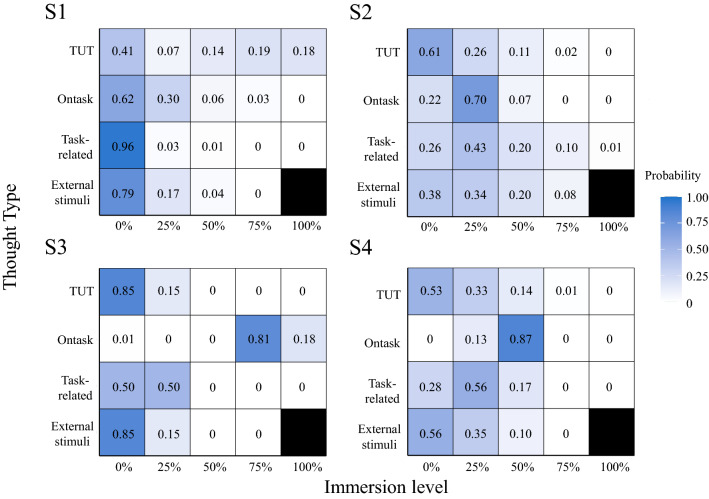
The estimated state by hidden Markov model. The vertical axis shows each type of thought, and the horizontal axis shows the degree of immersion. The participant’s report of 100% immersed in external stimuli-related thought is shown in black because it was not observed. Each color becomes darker as the probability increases. For example, the probability of selecting 1 (did not think about task-related thoughts at all) is very high in state 1. Each state was defined as TUT, unfocused, task-focused, and mixed.

HMM was used to estimate the attentional states behind self-reports to examine whether coexistence exists as a meaningful state of attention. The model with four states was used for subsequent analyses because the BIC was the smallest (Fig. 2A). The estimated states are shown in Fig. 3. The figure's vertical axis, horizontal axis, and color indicate thought type, immersion level, and probability, respectively. The figure shows the probability of selecting the immersion level for each thought type. For example, in State 1, the probability of selecting 0% to estimate task-related thought (i.e., the participant did not think about task-related thought) was very high. In State 1, only TUT immersion was higher than the other thought types, which can be construed as a TUT-tendency state. In the second state, immersion in all thought types was low, except for task-and external stimuli-related thoughts. We interpret this as an unfocused state. State 3 indicates that participants concentrated on the task, which could be construed as a task-focused state. The last was a state in which the highest probability of immersion in the task was 50%, and the likelihood that immersion in other thoughts was not zero, especially task-related thoughts. This state is interpreted as participants focusing on the task with other thoughts (interpreted as a mixed state of multiple thought types). We defined each state as TUT, nonfocused, task-focused, or mixed. Figure 2B,C show the estimated transition probabilities and occurrence rates of the states, respectively.

Among the thought response patterns during the intervals classified into each state, those that were particularly frequent are listed in Table 5. For example, in State 3, which is assumed to be task focused, responses to Ontask are often observed as four or five. In State 4, which is assumed to be a mixed state, the responses of three were observed for other thought dimensions in addition to Ontask. The thought patterns classified into each state are shown in S2–5 of the Supplementary Table.

**Table 5:** The representative patterns of responses at each state.

State number	Ontask	Task-related	External stimuli	TUT
State1	1	1	1	1
	1	1	1	5
	2	1	1	1
	1	1	2	4
	2	1	1	4
State2	2	2	2	2
	2	4	1	1
	2	3	2	1
	2	2	3	1
	2	2	1	3
State3	4	2	1	1
	5	1	1	1
	4	1	1	2
	4	1	2	1
	4	1	1	1
State4	3	2	2	1
	3	2	1	2
	3	3	1	1
	3	1	1	3
	3	1	3	1

The transition matrix indicates the probability that a particular state at one time point (t) changes to another or the same state at the next (t + 1). A transition to the same state can also be considered as continuing in this state. First, the transition probabilities were higher among the concentrated states in the task (between States 3 and 4) than among the others. Furthermore, the transition from the task-focused state to the TUT or unfocused state (States 1 and 2) and from the TUT state to the focused state was not frequent. Finally, the transition probabilities to the same state were exceptionally high in the TUT and unfocused states, indicating that these states are likely to be maintained.

To examine the behavioral tendency of the estimated states, we conducted a within-participants factor analysis using a one-way ANOVA on the RTV for each state. The results showed significant differences (F^2,26^ = 10.93, *p* < 0.001, η^2^ = 0.20). Subsequent tests using Tukey’s honestly significant difference test revealed that RTV in the TUT state was significantly greater than that in the mixed state (mean difference = 0.17, *p* < 0.001; mean difference = 0.16, *p* < 0.001). In contrast, there was no difference between the task and mixed conditions (Fig. 2D).

### Results of the hidden Markov model (RTV)

**Figure 4:**
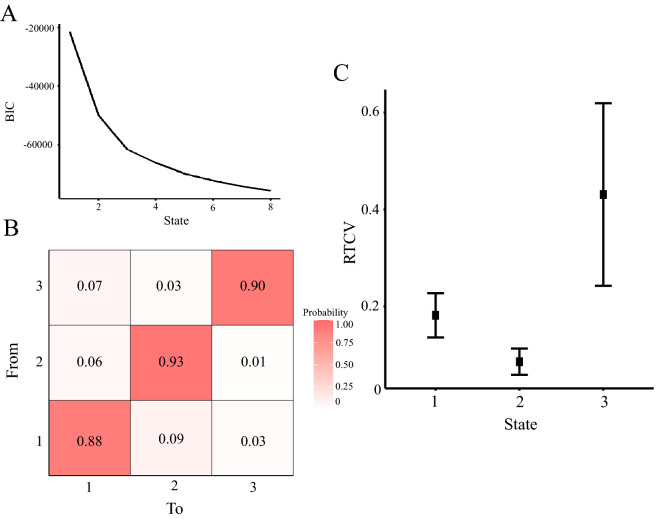
Results of applying the hidden Markov model to the time series of response time coefficient of variability (RTV). (**A**) The Bayesian information criterion (BIC) for each state. Since the BIC tends to decrease as the number of states increases, we used the elbow method to determine the number of states. Therefore, we estimated the BIC under the condition that the number of states was three. (**B**) Transition probability between states. The probability of transition from the state on the vertical axis to those on the horizontal axis is shown. (**C**) Estimated states. The horizontal axis shows the state, and the vertical axis shows the RTV. The darker the color, the higher the probability of transition. The points indicate the mean values, and the error bars indicate the standard deviations.

We investigated the relationship between separately estimated attentional states using self-reports and RTV. First, the HMM was applied to the time-series data of RTV to estimate the attentional states behind the data from the behavioral measurements. Because the BIC tended to decrease as the number of states to be estimated increased (Fig. 4A), we determined the number of states using the elbow method. The number of states was determined to be three because the decreasing trend of BIC decreased from this point (Fig. 4A). Estimated States 2 and 3 showed the smallest and largest mean and SD characteristics, respectively. However, State 1 was in between these two states and had no evident characteristics. The means and SDs for each state are listed in Table 6. Each state was defined as a middle steady, high steady, or behavioral MW state. The estimated transition probabilities between the states and their means and variances are shown in Fig. 4B,C, respectively.

**Table 6:** The mean RTV at each state was estimated using the hidden Markov model from behavioral indices.

	Mean	SD
S1	0.18	0.05
S2	0.09	0.03
S3	0.43	0.19

SD = standard deviation.

Next, we conducted a correlation analysis between the attentional state from self-reports and RTV. We used the index of total time spent by the participants in each state. There was a positive correlation (*r* = 0.46, *p* = 0.04) between TUT state and behavioral MW state. There was also a marginally significant negative correlation between behavioral MW state and mixed state (*r* = −0.41, *p* = 0.08). Detailed statistical values are provided in Table 7.

**Table 7:** Correlation coefficients between the total time of estimated states using self-report and response time variance.

	S1 (RTV)	S2 (RTV)	S3 (RTV)
S1 (self-report)	−0.05	−0.36	0.46*
S2 (self-report)	−0.07	0.04	0.06
S3 (self-report)	−0.08	0.29	−0.29
S4 (self-report)	0.28	0.14	−0.41

RTV = response time coefficient of variability; .05: "*".

## Discussion

This study aimed to investigate whether multiple thought types coexist during a task, using thought probes asking the immersion level of each thought type separately. We also estimated the attentional state behind the self-reports and behavioral variability. Most self-reports indicated the coexistence of different thought types. Moreover, the estimated states were task-focused, TUTs, unfocused, and mixed states of task-focused and other thoughts. These results suggest that individuals can allocate attention to more than one thought and discriminate and report it.

The number of self-reports is the simplest method of examining the coexistence of thought types. The number of responses in which the degree of immersion in a particular type of thought was 25% or more with no other thoughts was 79 out of the total number of probes (560 times). The probability that the participants immersed in a single type of thought was smaller than expected, and most of the thoughts were a mixture of multiple types. In addition to descriptive statistics, we estimated the attentional states to investigate coexistence among them. As the results of the HMM, there were the following states behind the self-reports: task-focused, TUT state, a low level of immersion in external stimuli and task-related thoughts (unfocused state), and task-focused thoughts with other thoughts (mixed state). In a study that used the HMM to estimate the attentional state behind self-reports, the participants’ reports were divided into three states: focused on the task, MW, and somewhat focused on the task and MW^26^. While we estimated similar states for task-focused and TUT, the intermediate state between task and MW was more complex, allocating attention to multiple thought types. The results show that the main state of attention during the task had the coexistence of thought types that could be separated from TUT and task concentration. Furthermore, it has been shown that unfocused and mixed states accounted for 58% of the task time in Fig. 2C. This study supports that the options to catch MW should include at least task-focused thoughts, task-related thoughts, and TUTs^5^.

There were several significant findings on transition probabilities concerning the HMM results obtained from the self-reports. First, the transition probabilities were higher between the task-focused and mixed states than the others. This result can be interpreted as task concentration, and mixed states did not last for a long time, and that concentration level changed frequently. Since the task used to measure TUTs was the SART demanding a low cognitive load, maintaining a high focus may be challenging. Furthermore, the relatively high number of transitions to the same state from TUT and unfocused state indicates that these thoughts are difficult to escape once they start. Previous research has reported a trend opposite to that observed in this study: the tendency for task concentration to persist more than MW concentration^25,26^. Since we estimated two task-related states from our data, the task-related state may become more stable than the TUT and unfocused states when these are integrated. It is also the case that we observed TUT and unfocused state less frequently than the task-focused state, which may have prevented us from ensuring variation in the thought transitions. Finally, the transition from a task-focused state to TUT or a unfocused state was less frequent, and the transition from TUT to a task-focused state was also less frequent. Prior research has proposed an off-focus state between the MW and task-focused states^35^. In this framework, MW is a state where participants focus on content unrelated to the task and is called the active MW. It was also proposed that this off-focus state is mediated between the MW and the task focus. We observed little transition between the task-focused and TUT states, and the transition between each thought occurred via the unfocused state in this study. Although the data are preliminary, these results suggest that the unfocused state interpreted the off-focus state and that the transition from the task-focused state to the other thought state may be made via the off-focus state. There are few transitions from high steady to behavioral MW, which also may support this hypothesis.

The states obtained by the HMM showed an association with response variability. Although there were significant differences in RTV between the TUTs state with the mixed state and the task-focused state, there was no significant difference between the mixed state and the task-focused state (Fig. 2D). In other words, the RTV changed between the mixed and MW states, but further concentration over the mixed state did not affect the RTV more. Maintaining a minimum level of task concentration (50% in this study) is considered sufficient to maintain task performance. Also, many studies have shown that the self-reports of MW were related to behavioral variance^23,25,31^. The estimated states’ validity was supported by behavioral indices, as the states associated with TUT have a larger RTV and the states associated with task concentration have a smaller RTV.

Some studies found that the depth of MW linearly increases the response time variance^26,29,36^. These studies used gradual options from fully concentrating to immersion in MW as the same dimension. In the other study, participants were asked about their intensity of focus on the task or TUT, respectively^37^. The results showed that RTV tended to decrease according to the level of immersion in the task, while for TUTs, RTV increased numerically but not significantly. In the present study, while there was a significant main effect of attention allocation to the task on RTV, this was not observed for TUTs. The results are inconsistent with the above studies, which predicted a linear relationship, while these are consistent with studies that examined the intensity of the task-focus and TUT separately. From these results, it is suggested that when the probes were separated, MW alone had little effect, and only task concentration may be related to task performance. The multiple regression results showed that immersion in the TUT harmed RTV when the participants were not focused on the task. While the task used in MW research was simple and demanded low cognitive resources, MW used various executive functions such as future thinking and autobiographical memory. These differences in the consumed resources for each thought may underline the multiple regression results. It is suggested that unfocusing on the task and focusing deeply on the TUT may harm RTV by not using resources for the task and other thoughts, respectively. In addition to the effect of focusing on the task, there was also an effect from using resources for other thoughts when not focused. These results also suggest the problem of considering MW and task concentration in the same dimension when examining the effect of MW on behavior.

The multiple regression analysis also revealed that task concentration significantly decreased RTV when immersed in TUT. Previous research and the HMM results of the present study indicate that TUT states are related to high RTV^25,31^. Our results suggest that even in states associated with high RTV, task-focus decreases the variability in reaction time. When immersion in TUT was high, performance was maintained when the rest of the attentional resources were allocated to the task. We propose that these mechanisms may explain the discrepancy between self-reports and behavior. If the person is immersed in MW but also divided some attentional resources to the task, the thought report will be labeled as MW in the conventional probes, but the behavior will be stable. Our results suggested that the resources not used by MW underlie the incomplete correspondence between MW and the behavior index. This result also highlights the advantage of our method, asking about the immersion level of each thought type.

The HMM was also applied to the time series of the RTV to examine how self-reports of thought relate to behavioral measures. We estimated the states with low RTV (high steady), high RTV, and high variance (behavioral MW), and slightly high RTV but low variance (middle steady state). Based on the previous study and this study’s results, the state with very high RTV can be interpreted as behavioral MW^25,27^, and a high steady state also can be construed as task-focused. The middle steady state has fewer characteristics than the other two. A positive correlation was found between MW states obtained from the self-reports and RTV. However, the correlation coefficients were moderate and not wholly identical or in a one-to-one correspondence. Furthermore, there was a negative correlation trend between behavioral MW and the mixed state estimated from the self-reports. The mixed state was the condition that occupied the longest time in this study (Fig. 2C). Although it should be noted that this is a trend, this result may indicate that maintaining a certain level of focus is more critical for behavioral MW in terms of task performance than fully concentrating on the task.

Studies using the MDES have focused on the coexistence of spontaneous thoughts and task focus. In these studies, PCA was used to estimate the common patterns in various self-reports^22–24^. It is essential to use each method to answer research questions. For example, the MDES method is suitable for exploring the content of *spontaneous thoughts* in detail, whereas our approach is suitable for examining *thought processes* in detail. Similarly, the conventional method has advantages when the participants' cognitive function is low, detailed classification is difficult, and the task time is limited. It is also possible to apply the HMM to the MDES. In the case of negative thoughts, such as ruminations, it may be difficult to change to other thought types immediately after the start. Transition patterns related to spontaneous thought content, such as in Zanesco (2020)^34^, are worth exploring.

Finally, we view the separation of the thought dimension and multiple tasks as limitations. The participants were asked to report their immersion levels in each thought dimension. When coexisting thoughts are reported, it is challenging to determine whether these are separate thoughts (multitasking) or whether multiple thought dimensions are observed in a single thought. The present study seems to indicate that different dimensions of off-tasks coexist to some extent and that their combination has different effects on behavioral indices. However, future studies are required to examine the simultaneity of thoughts in more detail.

Our study found that the participants simultaneously focused on multiple thought types. However, the meaning of simultaneity may differ depending on the time window in which participants report their thought content. Participants were required to recall the content of their thoughts in response to the probe. The extracted thoughts were not recorded before the probes. Some examples of participants’ reports from studies using descriptive experience sampling methods show that their thoughts frame variable time windows^38,39^. If the participants responded to our probes in the same way, there might have been two stories: they simultaneously allocated their attention to multiple thoughts or interpreted a series of different thoughts as a single thought. This study detected mixed states based on subjective reports; therefore, it was impossible to clarify whether attention was allocated to the thought content in parallel or serially. This ambiguity is also a problem with conventional probing methods. MW research has examined the behavioral indices and brain activity associated with MW for a few seconds or trials before the probe was presented. Brain activity can be misestimated if the most recent thought is reported in a serial thought sequence. Furthermore, even in the parallel case, the pure state could not be extracted because the participants labeled their thoughts as one thought type. Future studies should investigate whether such mixed states are parallel or serial using dynamic indices such as brain activity.

## Supplementary Information

Supplementary Information.

## Data Availability

The authors declare no competing interests.
