# Supplementary material for: Coexistence of thought types as an attentional state during a sustained attention task
Source: Sci Rep. 2023 Jan 28;13:1581. doi: 10.1038/s41598-023-28690-1 (PMC9884194; doi:10.1038/s41598-023-28690-1)
Supplement: Supplementary file 1 — Supplementary Information. [file 41598_2023_28690_MOESM1_ESM.pdf]

Coexistence of Thought Types as an Attentional State During a Sustained Attention Task

Kazushi Shinagawa*,[1,2] Yu Itagaki,[1] and Satoshi Umeda[1,3]

[1]Department of Psychology, Keio University

[2]Japan Society for the Promotion of Science

[3]Keio University Global Research Institute

Supplementary Table S1.
Frequency table of each response pattern to the thought probes.

Ontask	Task-related	External stimuli	TUT	Frequency
4	2	1	1	73
3	2	2	1	47
3	2	1	2	38
5	1	1	1	27
4	1	2	1	23
3	3	1	1	22
4	1	1	2	22
1	1	1	1	21
3	1	1	3	18
1	1	1	5	16
3	1	3	1	16
2	2	1	3	15
2	2	2	2	15
2	3	2	1	14
2	2	3	1	13
2	1	1	1	12
2	4	1	1	12
2	1	1	4	9
2	1	4	1	8
3	1	2	2	8
1	1	2	4	7
2	1	3	2	7
2	2	2	1	7
2	3	1	2	7
2	1	2	3	6
2	2	1	1	6
3	1	1	1	6
2	1	1	2	4
2	1	3	1	4
2	2	1	2	4

2	3	1	1	4
3	1	1	2	4
3	2	1	1	4
1	1	2	3	3
1	2	1	4	3
1	2	4	1	3
1	3	1	3	3
1	3	2	2	3
2	1	1	3	3
2	1	2	2	3
4	1	1	1	3
1	1	1	2	2
1	1	1	3	2
1	1	1	4	2
1	1	2	2	2
1	1	3	1	2
1	1	3	2	2
1	2	2	1	2
1	2	2	3	2
1	2	3	1	2
1	2	3	2	2
1	3	1	1	2
1	4	2	1	2
1	5	1	1	2
2	1	2	1	2
1	1	3	3	1
1	1	4	1	1
1	3	2	1	1
1	3	3	1	1
1	4	1	1	1
3	1	2	1	1

Supplementary Table S2.
Frequency table of each response pattern to the thought probes from State 1.

Ontask	Task-related	External stimuli	TUT	Frequency
1	1	1	1	18
1	1	1	5	16
2	1	1	1	10
1	1	2	4	7
2	1	1	4	6
2	1	1	3	3
2	1	2	3	3
3	1	1	1	3
1	1	1	2	2
1	1	1	3	2
1	1	1	4	2
1	1	2	2	2
1	1	2	3	2
1	2	1	4	2
2	1	1	2	2
1	1	3	1	1
1	1	3	3	1
1	3	1	3	1
2	1	2	1	1
2	1	2	2	1
3	1	3	1	1
4	1	1	1	1
4	1	2	1	1

Supplementary Table S3.
Frequency table of each response pattern to the thought probes from State 2.

Ontask	Task-related	External stimuli	TUT	Frequency
2	2	2	2	12
2	4	1	1	12
2	3	2	1	11
2	2	3	1	10
2	2	1	3	9
2	1	4	1	8
2	1	3	2	7
2	2	2	1	7
2	2	1	1	6
3	2	2	1	6
2	3	1	2	5
2	1	3	1	4
2	3	1	1	4
1	2	4	1	3
1	3	2	2	3
2	2	1	2	3
1	1	1	1	2
1	1	3	2	2
1	2	2	1	2
1	2	2	3	2
1	2	3	1	2
1	2	3	2	2
1	3	1	1	2
1	3	1	3	2
1	4	2	1	2
1	5	1	1	2
2	1	1	1	2
2	1	1	2	2
2	1	2	2	2
2	1	2	3	2

3	2	1	2	2
1	1	2	3	1
1	1	3	1	1
1	1	4	1	1
1	2	1	4	1
1	3	2	1	1
1	3	3	1	1
1	4	1	1	1
2	1	1	4	1
2	1	2	1	1
3	3	1	1	1

Supplementary Table S4.
Frequency table of each response pattern to the thought probes from State 3.

Ontask	Task-related	External stimuli	TUT	Frequency
4	2	1	1	73
5	1	1	1	27
4	1	1	2	22
4	1	2	1	22
4	1	1	1	2
1	1	1	1	1

Supplementary Table S5.
Frequency table of each response pattern to the thought probes from State 4.

Ontask	Task-related	External stimuli	TUT	Frequency
3	2	2	1	41
3	2	1	2	36
3	3	1	1	21
3	1	1	3	18
3	1	3	1	15
3	1	2	2	8
2	2	1	3	6
3	1	1	2	4
3	2	1	1	4
2	2	2	2	3
2	2	3	1	3
2	3	2	1	3
3	1	1	1	3
2	1	1	4	2
2	3	1	2	2
2	1	2	3	1
2	2	1	2	1
3	1	2	1	1
